# The Treatment of Multiple Myeloma Patients Not Eligible for Asct

**DOI:** 10.4084/MJHID.2010.009

**Published:** 2010-05-03

**Authors:** Paul Richardson, Jacob Laubach, Anuj Mahindra, Constantine Mitsiades, Robert Schlossman, Irene Ghobrial, Teru Hideshima, Noopur Raje, Nikhil Munshi, Kenneth Anderson.

**Affiliations:** Jerome Lipper Multiple Myeloma CenterDana-Farber Cancer Institute and Massachusetts General Hospital, Harvard Medical School, Boston, Massachusetts

## Abstract

Advances in therapies for younger patients with multiple myeloma have resulted in significant improvements in outcome over recent years, on the contrary the progress in treatments for elderly patients has remained more modest. Traditionally, patients who are not eligible for transplantation, like the older patients, have been treated with the combination of melphalan plus prednisone (MP), which leads to responses in approximately 50% of patients; however, patients rarely achieve a complete response (CR) and long-term outcomes are disappointing, with a relapse-free survival of approximately 18 months and an overall survival (OS) of approximately 3 years.

With the arrival of novel agents, including the first–in-class proteasome inhibitor, bortezomib, and the immunomodulatory agents, thalidomide and lenalidomide, a shift in the management of older patients and/or those not eligible for transplantation has taken place. Increasingly, novel agents are now being incorporated into therapy, based on the positive findings from clinical trials in this setting, and outcomes have improved accordingly.

Whereas advances in therapies for younger patients with multiple myeloma have resulted in significant improvements in outcome over recent years, progress in treatments for elderly patients has remained more modest.^1^ Traditionally, patients who are not eligible for transplantation have been treated with the combination of melphalan plus prednisone (MP), which leads to responses in approximately 50% of patients; however, patients rarely achieve a complete response (CR) and long-term outcomes are disappointing, with a relapse-free survival of approximately 18 months and an overall survival (OS) of approximately 3 years.^2^

With the arrival of novel agents, including the first–in-class proteasome inhibitor, bortezomib, and the immunomodulatory agents, thalidomide and lenalidomide, a shift in the management of older patients and/or those not eligible for transplantation has taken place. Increasingly, novel agents are now being incorporated into therapy, based on the positive findings from clinical trials in this setting, and outcomes have improved accordingly. 3

Specifically, a number of studies have investigated the addition of novel agents to the traditional MP regimen. The combination of MP plus thalidomide has been investigated in five randomized trials.^4^–^9^ In all studies, the addition of thalidomide to MP resulted in a significant improvement in overall response rate (ORR) and CR rates, as well time to progression (TTP), progression-free survival (PFS) or event-free survival (EFS) (**Table 1**). A significant benefit in terms of OS, however, was only seen in the two studies conducted by the Intergroupe Français du Myélome [IFM] (*P*=0.0006, *P*=0.03).^4^,^5^ The most frequent grade 3/4 adverse events reported included hematological toxicities, thromboembolism, infections, and gastrointestinal side-effects.^4^,^6^

Thalidomide has also been combined with dexamethasone in a trial conducted by Ludwig *et al*.^10^ evaluating elderly patients with newly diagnosed multiple myeloma. Compared with MP, thalidomide plus dexamethasone (TD) resulted in higher ORR (68% versus 50%, *P*=0.0023) and CR plus very good partial response (VGPR) rates (26% versus 13%, *P*=0.0066). TTP (21.2 versus 29.1 months, *P*=0.2) and PFS (16.7 versus 20.7 months, *P*=0.1) were similar in both arms. However, MP proved superior to TD in terms of OS (49.4 versus 41.5 months, *P*=0.024). Notably, the number of early deaths within the first year was significantly higher in the TD arm (28% versus 16%, *P*=0.014). In addition, TD resulted in a higher incidence of toxicity, which was observed particularly in patients older than 75 years and those with poor performance status.

Lenalidomide has also been studied in the elderly population. A Phase I/II trial by Palumbo *et al*.^6^ which investigated the combination of lenalidomide with MP in elderly patients with newly diagnosed multiple myeloma yielded positive results, with impressive ORR and a favourable side effect profile. These data require confirmation in randomized clinical trials, and a number are ongoing, with results anticipated soon. In addition, data from the randomized ECOG EA403 study comparing lenalidomide with high dose dexamethasone (RD), versus lenalidomide with low dose dexamethasone (Rd), have shown efficacy in older patients, with promising ORR, PFS and OS but significant toxicity with the higher dose dexamethasone such that OS proved inferior with RD compared to Rd, confirming the importance of using relatively steroid-sparing approaches in this population.^7^

The combination of bortezomib with MP (VMP) has been explored in the large Phase III VISTA trial and was found to be significantly superior to MP in terms of ORR and CR rates, TTP, and 3-year OS.^11^,^12^ The ORR, determined using the stringent European Group for Blood and Marrow Transplantation criteria, was 71% with VMP compared with 35% with MP, with an immunofixationnegative CR rate of 30% with VMP versus 4% with MP (*P*<0.001). TTP was significantly longer in the VMP arm than in the MP arm (24 months versus 16.6 months, *P*<0.001). Although median OS was not reached in either arm after a median follow-up of 25.9 months, VMP demonstrated a significantly superior 3-year OS compared with MP: 72% with VMP versus 59% with MP (*P*=0.0032). Fewer patients in the VMP versus MP arm required subsequent therapy (38% versus 57%). The time to next therapy was 28.1 months for VMP versus 19.2 months for MP (*P*<0.000001). In addition, patients receiving VMP had a significantly longer treatment-free interval (TFI) compared with those receiving MP (16.6 versus 8.4 months, *P*<0.000001). Subanalyses of the VISTA study showed that VMP remains effective in patients with renal impairment, in those with cytogenetic abnormalities, and that the concomitant use of erythropoiesis-stimulating agents does not negatively impact on PFS and OS or increase the risk of thromboembolic events.^11^–^14^

The main differences in the incidence of grade 3/4 adverse events between the VMP and MP arms were seen for gastrointestinal side effects, peripheral neuropathy (PN), and herpes zoster infection, which were found to be more frequent in the VMP arm, with the latter proving readily manageable with anti-viral prophylaxis. PN grade 3 was observed in 13% of patients, with grade 4 PN observed in <1% of patients receiving VMP. However, PN was reversible in most patients; 79% of PN events improved (≥ 1 grade) in a median of 1.9 months and 79% of PN events completely resolved in a median of 5.7 months.

The VISTA trial demonstrated that VMP is significantly superior to MP in terms of TTP (*P*<0.001), CR (*P*<0.001), ORR (*P*<0.001), TFI (*P*<0.000001), and OS (*P*=0.0032). These data have significant implications for the treatment of patients with newly diagnosed disease who are not eligible for transplantation, including those with high risk disease; results from this controlled trial show that VMP should be considered a new standard of care for these patients and provided the basis for FDA and EMEA approval for the use of bortezomib in the upfront setting in 2008.

Two ongoing studies in the elderly population are currently investigating reduced bortezomib dose intensity in combination with MP. Instead of the twice-weekly dose, bortezomib is administered once weekly. Early results indicate that significant efficacy is maintained with the less frequent bortezomib schedule (**Table 2**), while tolerability is increased substantially. Notably, grade 3/4 PN was only 2% or 5% with the reduced dose VMP regimen in the two studies.^15^,^16^ Moreover, the rate of treatment discontinuations was low in both studies (8% and 10%).^15^, ^16^ Although longer follow-up is needed to assess PFS and OS, the results suggest that bortezomib administered once weekly in combination with MP is effective in elderly patients with increased tolerability, suggesting that this may be a particularly useful regimen in patients who cannot tolerate the full-dose VMP regimen, such as very elderly or frail patients.

Combinations of novel agents, informed by preclinical studies^17^, have also been studied in older patients as part of Phase I/II trials, with promising results to date^18^. Specifically, the combination of lenalidomide, bortezomib and dexamethasone (so called RVD) has shown an ORR of 100% with a VGPR of 74% and nCR/CR of 44%, with patients up to the age of 86 years included, and a substantial portion over the age of 70. No treatment mortality has been reported and toxicities have proven manageable, with low rates of both significant PN (3%) and DVT (5%) seen. Moreover, responses have been durable, with activity in high risk disease also noted. 18

In spite of well documented improvement in patient outcomes associated with the introduction of novel agents in multiple myeloma 19, nearly all patients relapse and require additional therapy. As is true for newly diagnosed multiple myeloma, novel agents have assumed an increasingly important role in the management of patients with relapsed and refractory disease.

Following a series of encouraging phase I/II clinical trials 20, 21, the efficacy of lenalidomide in relapsed and refractory MM was unequivocally demonstrated in two large, phase III trials comparing lenalidomide plus dexamethasone to lenalidomide plus placebo, the MM-009 22 and MM-010 23 studies. The median age of patients treated with lenalidomide and dexamethasone was 64 in MM-009 and 63 in MM-010, while the median age of those in dexamethasone arm was 62 and 64, respectively. However, both studies included a substantial portion of elderly patients. In both studies, lenalidomide plus dexamethasone was superior to dexamethasone plus placebo in terms of OR (61% versus 19.9%, P < 0.001 in MM-009 and 60% versus 24%, P < 0.001 in MM-010), CR (14.1% versus 0.6%, P < 0.001 in MM-009 and 15.9% versus 3.4% in MM-010), TTP (11.1 months versus 4.7 months, P < 0.001 in MM-009 and 11.3 months versus 4.7 months, P < 0.001 in MM-010), as well as OS (29.6 months versus 20.2 months, P < 0.001 in MM-009 and median OS not reached versus 20.6 months in MM-010, with hazard ratio for death 0.66). Grade 3/4 toxicities were more common with lenalidomide and dexamethasone, particularly neutropenia and venous thromboembolism. Based on these studies, lenalidomide in combination with dexamethasone has received approval from both the FDA and EMEA for treatment of relapsed and refractory multiple myeloma.

Bortezomib is also an effective treatment strategy in patients with relapsed multiple myeloma. This was suggested by phase I/II trials 24,25,26 and confirmed in a randomized phase III study wherein patients received either bortezomib or high-dose dexamethasone) 27. The median age was 62 in the bortezomib arm and 61 in the high-dose dexamethasone arm, an age distribution reflecting the age demographics of multiple myeloma with again a proportion of older patients included. Bortezomib outperformed high dose dexamethasone in terms of OR (38% versus 18%, P < 0.001), CR (6% versus 1%, P < 0.001), TTP (6.22 months versus 3.49 months, P < 0.001), and one-year survival rate (80% versus 66%, P = 0.003). In an updated analysis including final time to event data, the OR and CR rates in the bortezomib arm of this trial were 43% and 9% 28. The median survival for bortezomib-treated patients in this analysis was 29.8 months versus 23.7 months in the dexamethasone group. Bortezomib was associated with a higher rate of grade 3/4 toxicities (75% versus 60%). PN was more common with bortezomib (36% versus 9%), although in most instances PN was grade < 2 and reversible with suggested dose modification or treatment discontinuation. Thrombocytopenia occurred in 35% of bortezomib-treated patients versus 11% among those who received dexamethasone, but was cyclical with platelet count recovery during the 10-day rest period and not associated with an increased incidence of significant bleeding events. The incidence of herpes-zoster reactivation was also higher in the bortezomib arm (13% versus 5%; P < 0.001), confirming the need for -antiviral prophylaxis in these patients.

As in the setting of newly diagnosed disease, regimens involving combinations of novel agents are undergoing evaluation in relapsed multiple myeloma and have produced promising results to date. In a phase I/II study involving 85 patients with advanced disease, bortezomib, thalidomide, and dexamethasone (VTD) yielded an OR rate (minimal response [MR] or greater) of 79% and a nCR rate of 22% 29. RVD has also been evaluated in refractory multiple myeloma; in a phase II study involving 63 patients, the combination was an associated with at least an MR in 86%, a PR or better in 67%, and a nCR or better in 24%) 30. The regimen has been well tolerated with only one episode of grade 3 PN, very rare DVT and primarily grade 1-2 myelosuppression.

Although the management of multiple myeloma in older patients not eligible for transplantation provides considerable challenges, there is now reason for greater optimism. A range of novel agent combinations are available which have demonstrated superior efficacy over the traditional combination chemotherapy, such as MP, indicating that MP should no longer be considered the standard of care in this population. Ongoing studies will establish optimal dosing and treatment schedules for different populations, with the aim of maximizing rate and frequency of response, durability of remission and improving tolerability, especially in elderly and/or more frail patients. Future trials will also evaluate the integration of newer agents currently under development in the advanced setting (please see **figure 1**), with the goal of further improving patient outcome, as well as establishing doses and schedules in older patients associated with better efficacy in this particular population.

## Figures and Tables

**Figure 1:**
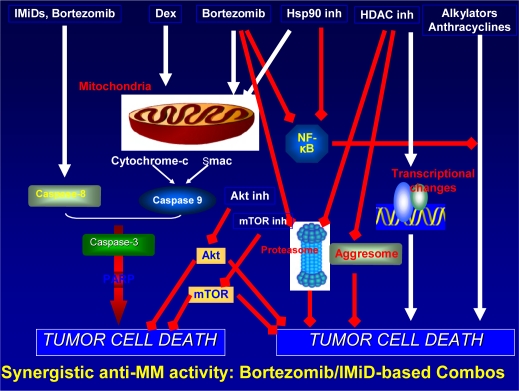


**Table 1:** Summary of five MPT Phase III trials conducted in the upfront setting.

**Regimen**	**n**	**CR+PR (%)**	**CR (%)**	**PFS/EFS/TTP**	**OS**	**Reference**
Thal/MP vs MP	129 126	76 48	16 4	21.8 m 14.5 m	45 m 47.6 m	Palumbo *et al. Blood* 2008; 112:3107–3114^5^
Thal/MP vs MP	191 124	76 35	13 2	27.5 m 17.8 m	51.6 m 33.2 m	Facon, *et al. Lancet* 2007; 370:1209–1218^3^
Thal/MP vs MP (>75 y)	113 116	62 31	7 1	24.1 m 19 m	45.3 m 27.7 m	Hulin, *et al. Blood* 2007;110 (Abstract 75)^4^
Thal/MP* vs MP	363	42 28	6† 3†	20 m 18 m	29 m 33 m	Gulbrandsen *et al. Haematologica* 2008;93 (Abstract 209)^6^
Thal/MP vs MP	152 149	66 47	2 2	EFS 13 m vs 9 m PFS 14 m vs 10 m	37 m 30 m	Wijermans *et al. Blood* 2008;112 (Abstract 649)^7^

* Thal doses: 200–400 mg.

† CR + near CR

**Table 2:** Bortezomib Phase III trials in upfront setting

**Regimen**	**n**	**CR+PR (%)**	**CR (%)**	**PFS/EFS/TTP**	**OS**	**Reference**
VISTA: VMP vs MP	337 331	71 35	30 4	24 m 16.6 m	3-year OS: 72% 59%	San Miguel *et al. NEJM* 2008; 359:906–917^9^
GIMEMA: VMPT vs VMP	177 177	87 82	39 21	2-year PFS: 84% 76%	3-year OS: 90% 89%	Palumbo IMW 2009 (Abstract 117)^14^
PETHEMA/GEM: VMP vs VTP	130 130	81 81	22 27	2-year PFS: 72% 65%	2-year OS: 88% 93%	Mateos IMW 2009 (Abstract 154)^13^
